# “They don’t like us….”: Barriers to antiretroviral and opioid substitution therapy among homeless HIV positive people who inject drugs in Delhi: A mixed method study

**DOI:** 10.1371/journal.pone.0203262

**Published:** 2018-08-30

**Authors:** Samresh Kumar, Himanshu A. Gupte, Petros Isaakidis, J. K. Mishra, Joseph Francis Munjattu

**Affiliations:** 1 India Health Action Trust (IHAT), Technical Support Unit, Delhi, India; 2 Narotam Sekhsaria Foundation, Mumbai, India; 3 Médecins Sans Frontières, Operational Research Unit, Luxembourg City, Luxembourg; 4 Delhi State AIDS Control Society (DSACS), Delhi, India; 5 India Health Action Trust (IHAT), Technical Support Unit, Bengaluru, India; Centers for Disease Control and Prevention, UNITED STATES

## Abstract

**Background:**

Provision of Anti-Retroviral Therapy (ART) and Opioid Substitution Therapy (OST) are important components of the targeted intervention (TI) programme for people who inject drugs (PWID). Homeless HIV positive PWIDs in Delhi is a key population experiencing gaps in uptake of these services, especially the ART uptake which is reportedly far from 90%, UNAIDS’ 90-90-90 target to end the AIDS epidemic.

**Objective:**

To assess the gaps and barriers in accessing the ART and OST services uptake among HIV positive homeless PWID in Delhi and to explore experiences and perspectives of the PWIDs and service providers.

**Methodology:**

We used a convergent parallel mixed methods design which included a cross-sectional quantitative survey and a qualitative study. Two hundred thirty five homeless HIV positive PWID were interviewed and in-depth interviews were conducted with five PWIDs and nine health providers.

**Results:**

While only 12% of PWIDs were on ART, 80% were availing OST services. The top individual, health system related and structural barriers for ART service access were insufficient and incorrect knowledge (63%), long waiting time (86%) and lack of family support (44%) respectively. Inconvenient timings, stringent registration requirements and negative attitude of health providers were expressed as major barriers of accessing ART services during the interviews while these were not a concern in OST services. Homelessness, poverty, stigma were common barriers for both services. Integrated, ‘single window’ service and provision of additional support like nutrition and shelter were suggested as measures to improve access by both health providers and the PWIDs themselves.

**Conclusion:**

There is an urgent need for structural and health systems changes to improve access to ART and OST services. These include integrated service delivery, flexibility in timing of the centers, accelerated ART initiation, simplification of bureaucratic procedures, nutritional and social support to all homeless HIV positive PWIDs.

## Introduction

Injecting Drug Use (IDU) is one of the most important routes of transmission for HIV. The risk of HIV acquisition among people who inject drugs (PWID) is 24 times higher than among adults (aged 15 years and older) in the general population [1]. Globally, PWIDs account for 7% of new HIV infections [1]. In India the current number of PWID is estimated to be 177,000 [2]. While a decline in prevalence has been achieved in North Eastern Indian States which have been the epicenter of the HIV epidemic among PWID in the country, newer pockets of high HIV prevalence among PWID have emerged over the past few years in other States, and particularly in large urban centers like Delhi and Mumbai [3].

As of June 2016, globally, 18.2 million people living with HIV were accessing ART, up from 15.8 million in June 2015 and 7.5 million in 2010 [4]. A global review of HIV prevention, treatment and care services for PWID has shown that only 4 per cent of the HIV-positive PWID (range 2%–18%) receive ART [5]. Similar to the global patterns, the latest National Integrated Biological and Behavioral Survey data, revealed that in Delhi the HIV prevalence among PWID was 22% (versus 10% at the national level) while only 67% of the HIV-infected PWID were linked to ART centers [6–7].

A major component of a targeted intervention programme for PWID is OST which is the administration of a legal, safer, long-acting agonist medication in combination with psychosocial rehabilitation. The benefits of OST are that it reduces risk behaviors and crime, drug consumption, the risk of lethal overdose and the risk of HIV transmission [7]. As of March 2017, about 217 OST centres in more than 100 districts of the country were functional covering about 21,000 PWID [8]. In Delhi, 1264 out of 2973 expected PWID were availing OST with a retention rate of 43% during the years 2015–16 [7].

PWIDs in Delhi is a critical key population experiencing a large coverage gap of ART and OST services and consequently characterized by an abysmal uptake of life-saving interventions, especially ART [7]. The proportion of PWID who are on ART is far away from the UNAIDS’ 90-90-90 ambitious treatment target to help end the AIDS epidemic [9]. Several barriers in accessing ART, OST and other services like attitude of health care providers, homeless PWIDs, confidentiality issues and financial problems etc. have been reported in various studies [10–12]. There is data which indicates the proportion of PWIDs on OST and ART. However, the highly vulnerable sub-population of homeless PWIDs has not been previously studied. Moreover, there is a gap in the coverage of OST and ART as per the field observations and interactions but there is no evidence of the same to understand the magnitude of the gaps which could help in developing strategies to address the same. This mixed methods study aimed to assess the gaps and barriers in OST and ART uptake, to explore patients’ and providers’ experiences and perspectives and to provide suggestions for improving the uptake and efficiency of these services among homeless HIV positive PWIDs.

## Materials and methods

### Study design

We used a convergent parallel mixed methods design which included a cross-sectional quantitative survey and a qualitative study (in-depth interviews) [13].

### Setting

#### General setting

Delhi is one of the world’s largest cities with a population of around 17 million [14]. The prevalence of HIV in the antenatal care in Delhi as per HIV sentinel surveillance 2014–15 report was 0.25 percent [15]. High risk populations for HIV: Female Sex Workers, Men having Sex with Men, PWID and transgenders are estimated to be approximately 83,500, as per site validation done in 2014–15 [7]. As part of the National AIDS Control Programme (NACP) strategy to reach out to key populations, the Targeted Intervention (TI) project provides specific prevention and treatment services for each population. In Delhi currently there are 81 TI projects which include 32 for Female Sex Workers, 11 for Men having Sex with Men, 15 for PWID, 6 for transgender, 13 for migrants and 4 for truck drivers.

#### Specific setting

The Targeted Intervention Project for PWID provides health education, abscess management, treatment referrals and provision of harm reduction services such as Needle and Syringe Exchange Programme, OST and ART. The services are implemented by Non-Governmental Organizations (NGOs), public service providers and executed through peer-based outreach, Drop-in Centres (DIC) and referrals and networking-based approaches. The TI project provides HIV prevention services to the high risk population whereas the OST centres at the government setup and at some of the TI project, provides OST drugs to the PWIDs. ART centres which are situated only at the government hospitals provide ART treatment to the positive PWIDs.

9400 PWID are covered under the 15 targeted intervention programs in Delhi. 80% of them are homeless and live on the streets without any family support. 8% HIV positivity was reported in the program data during 2015–16 [7].

#### Study site

The study was carried out in all 15 PWID TI projects in Delhi which were being implemented by NGOs, covering 9 public ART centers and 11 OST centers (4 NGO and 7 Public run).

#### Study population

For the quantitative questionnaire survey, we included all homeless PWID who were HIV-positive and active in the TI project as on December 2016. Active PWID were defined as PWID who availed at least one service of the TI programme within a six month period prior to the study.

For the qualitative study a subgroup of PWID and service providers of the NGOs, ART and OST centers, were interviewed to explore experiences and perspectives about access to ART and OST care. Interviewees of both groups were selected to meet the following inclusion criteria:.

**Inclusion criteria:**
1. HIV positive homeless PWIDs, above 18 years of age and registered under the TI program in Delhi.
2. Service providers of the NGOs, ART and OST centers who were currently in service at the respective centers.
3. HIV positive homeless PWIDs and service providers who were vocal and gave individual consent to participate in the study were purposively selected.

For quantitative data, we have covered all the HIV positive PWIDs registered under the Targeted Intervention program in Delhi. 299 HIV positive PWIDs are registered with the TI out of which 235 were available in the field and interviewed for collecting quantitative data. The sample constitutes a full assessment of homeless HIV positive PWIDs registered with TI in Delhi.

### Data variables, sources of data and data collection

**Fig 1:**
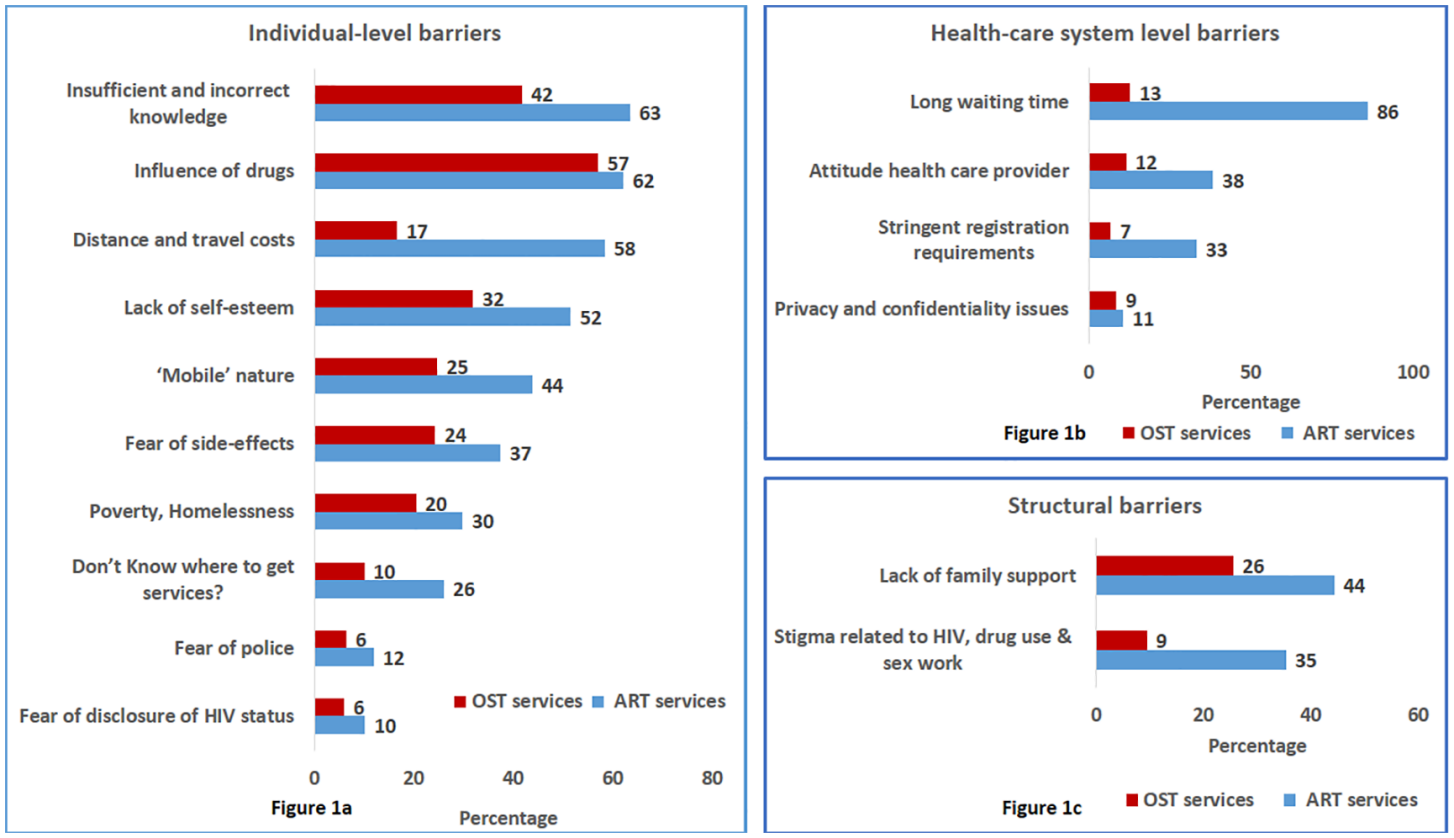
Barriers for accessing the ART and OST services among homeless HIV positive PWID registered in Targeted Intervention project in Delhi (December 2016).

A list of all eligible PWID who were HIV-positive was prepared based on the information collected and captured by the TI NGOs (People Living with HIV [PLHIV] line listing). The questionnaire, based on a similar study by the United Nations Office of Drug and Crime (UNODC) was validated and pilot tested before the actual data collection [11]. Additional options were also added to the list as per the results of the pilot. Ranking was done based on the cumulative responses by the PWIDs during analysis. The structured questionnaire contained multiple-choice questions about variables related to socio-demographic characteristics, drug use behavior, ART and OST service uptake. The barriers to accessing ART and OST services were captured through yes/no questions by listing of probable barriers. Barriers listed in the questionnaire included individual level barriers such as insufficient and incorrect knowledge of OST and ART, influence of drugs, distance and travel cost etc., healthcare system level barriers like long waiting time, attitude of health care providers, stringent registration requirement etc and structural barriers which included lack of family support and stigma related to HIV, drug use and sex work. (Details in Fig 1) The Principle Investigator (SK) did the record review and personally administered the structured data collection questionnaire to all eligible homeless HIV-positive PWID.

SK also interviewed the TI NGO staff (Project manager, counselor, and outreach worker), ART center staff (Doctor and counselor), OST center staff (Doctor and counselor) in their respective health facilities and the purposively selected PWID who met the inclusion criteria. Interviews with the selected PWID were conducted at DICs of the TI project. The interview guide was a validated tool from another UNODC report [12]. The interviewees from both groups were selected by aiming at maximum variation in representativeness of perceptions and experiences.

### Analysis and statistics

#### Quantitative

Data from the administered questionnaire were double-entered, validated and analyzed using EpiData version 3.1 for entry and version 2.2.2.182 for analysis (EpiData Association, Odense, Denmark). Descriptive statistics was used to summarize the quantitative data. Categorical variables such as socio-demographic characteristics and barriers to ART and OST services were summarized as proportions.

#### Qualitative

The interview data were transcribed and coded and manual thematic analysis was applied to the transcripts. Codes and themes were reviewed by two additional investigators (PI & HG) to reduce bias and increase credibility. The decision on coding rules and theme generation was done by using standard procedures and in consensus. Any differences were resolved by discussion. The findings were reported by using the ‘Consolidated Criteria for Reporting Qualitative Research [16].

#### Ethics

Ethics approval was obtained from the India Health Action Trust, Bangalore, India, and the Ethics Advisory Group of the International Union Against Tuberculosis and Lung Disease (The Union), Paris, France. For the quantitative component of the study, which involved retrospective review of TI project records, a waiver for informed consent was obtained from the ethics committees. Written informed consent was obtained for the stakeholder and PWID interviews. The consent form had two parts: information for the participant and the actual consent form, which was signed by the participant. An additional consent for audio recording of the interview was also obtained.

## Result

### Quantitative

#### PWID characteristics and uptake of OST and ART services

Out of total 299 homeless HIV positive PWID registered in the TI project, 235 were interviewed. Approximately half of them were in the age group 25–34 years and 61% were Illiterate. One third of them were daily-wage labourers, one-third were scrap metal/rag/garbage collectors while 11% were unemployed.

More than one third of PWID first injected drugs when they were less than 18 years old and 43% first injected drugs between 18–24 years. Although, the PWIDs covered under the study were all homeless, they lived with their friends, partners, night shelters (provides shelter only at night), outside closed shops, public parks or in the streets.

Mobility and needle sharing were frequent among the study participants with 56% having travelled out of Delhi at least once in last 30 days, and 63% of them having shared needle/syringe with other PWID in the place they visited last.

Overall, one in five PWID were not registered for OST and one in four were not registered in an ART center; only 12% of them were on ART while 63% were on pre-ART (Positive PWIDs on pre ART are those who are infected with HIV but their CD4 count is more than 500 and they do not require immediate initiation into ART) (Table 1).

**Table 1:** Demographic profile and uptake of Anti-Retroviral treatment and opioid substitution therapy services of the interviewed homeless HIV positive PWIDs registered in targeted intervention project in Delhi (December 2016).

Variable	Number of PWID	% of PWID
Total	235	(100)
**Age**		
18–24 Yrs	49	(21)
25–34 Yrs	104	(44)
35–44 Yrs	66	(28)
> = 45 Yrs	16	(7)
**Education**		
Illiterate	144	(61)
Literate	91	(39)
**Occupation**		
Daily wage labourer	76	(32)
Scrap/Garbage collection/ Rag picking	72	(31)
Unemployed	26	(11)
Service (private/government)	38	(16)
Drug dealer, Petty crime	23	(10)
**Marital status**		
Never married	159	(68)
Separated	38	(16)
Currently married	36	(15)
Widower/Divorced	2	(1)
**With whom do you currently live**		
Living alone	76	(32)
Living with friends	75	(32)
Living with spouse/ relative/ sexual partner	46	(20)
Rain Basera (Night Shelter)	38	(16)
**Which state do you belong/native state**		
Uttar Pradesh	116	(49)
Delhi	40	(17)
Bihar	27	(12)
Others	52	(22)
**PWID mobility (travelled out of Delhi/NCR in last 30 days)**		
Travelled	132	(56)
Did not travel	103	(44)
**PWID needle/syringe sharing status with other injecting drug users in the place visited last**		
Shared	83	(63)
Not shared	49	(37)
**PWID age group first injected drugs**		
5–12 Yrs	8	(3)
13–17 Yrs	76	(32)
18–24 Yrs	101	(43)
> = 25 Yrs	50	(21)
**Drug most often injected over the last 3 months**		
Buprenorphine (Tidigesic, Lupigesic, Morphine, Bupin etc.)	115	(49)
Brown Sugar/ Smack	55	(23)
Diazepam/ Calmpose, Nitrazepam/ Clonazepam/ Avil/ Phenargan Pethidine	33	(14)
Others	3	(1)
Currently not injecting	29	(12)
**Feel treated differently in health facilities/ hospitals**		
Yes	102	(43)
No	133	(57)
**PWID current ART status during the interview**		
Pre ART	149	(63)
On ART	29	(12)
Not registered at ART Center	55	(23)
Not recorded	2	(1)
**PWID current OST Status during the interview**		
Registered at OST Center	188	(80)
Not Registered at OST Centre	47	(20)

#### Barriers to ART and OST services

Fig 1 shows the different barriers faced by homeless, HIV-positive PWID that prevented them from accessing ART and OST services as well from being retained in care after enrollment. One fourth of the participants did not know where to get ART services, while 63% had insufficient and incorrect knowledge about ART. Active drug use (‘high’) (62%), distance and travel costs (58%), high mobility (44%) and poverty and homelessness (30%) were the main individual barriers to accessing ART services. For OST services the barriers at the individual level were similarly ranked (Fig 1a). Regarding health care system level barriers (Fig 1b), they were mostly reported for ART services and long waiting time was reported as the major barrier (86% of the PWID). Regarding structural barriers (Fig 1c) 44% and 26% expressed lack of family support related to ART and OST access respectively, while more than one in 3 PWID expressed stigma related to HIV drug use and sex work as important structural barriers to accessing ART services.

### Qualitative findings

In-depth interviews were conducted with 5 PWID and key informant interviews were conducted with 9 health providers (1 ART Medical Officer, 1 OST Medical Officer, 1 ART counsellor, 1 OST counsellor, 3 TI Project Managers, 1 TI counsellor and 1 outreach worker). The interviews lasted for an average duration of 25 minutes (range: 15 to 40 minutes).

**Fig 2:**
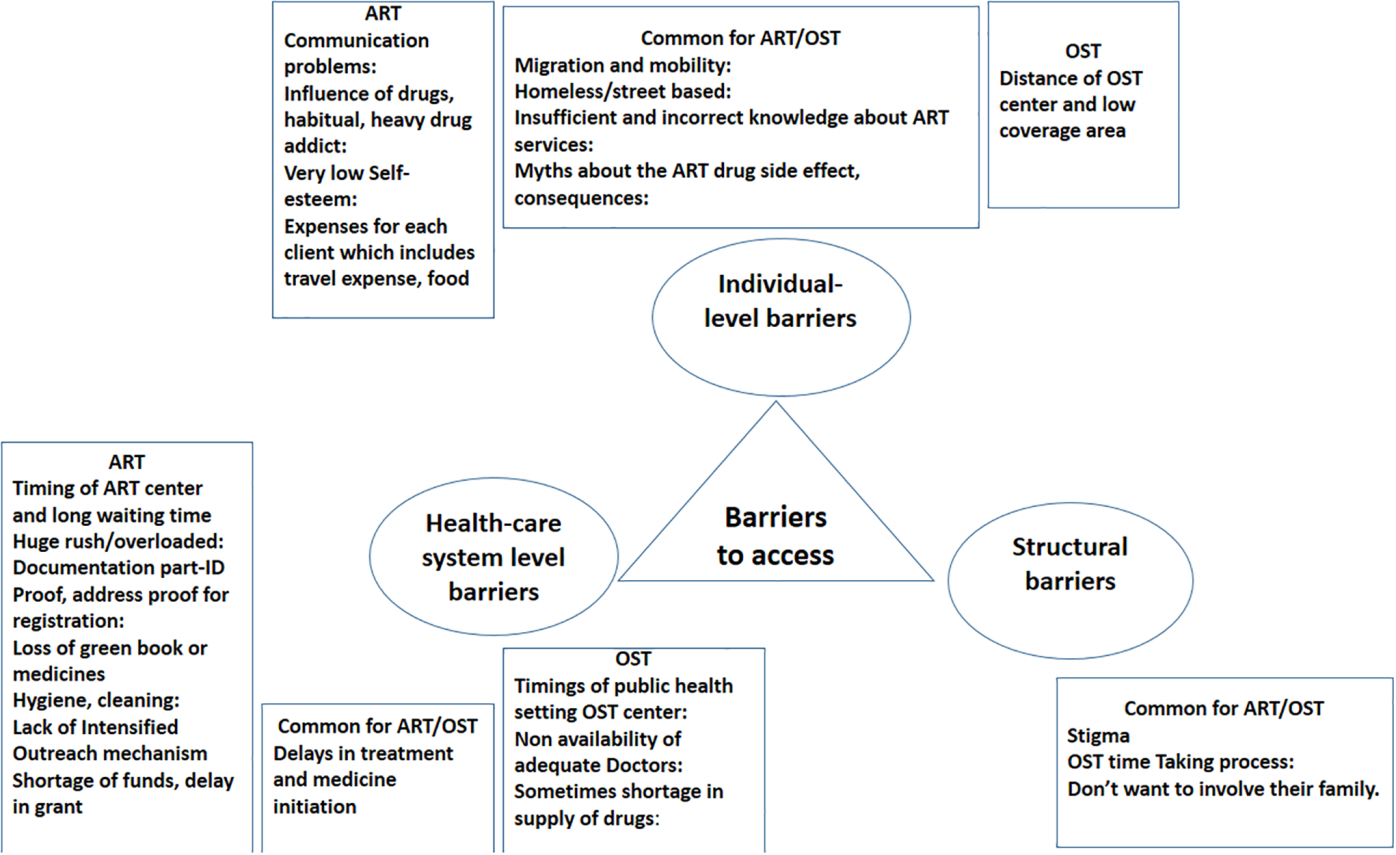
Codes and categories of barriers to accessing ART and OST services among homeless HIV positive PWID in Delhi (December 2016).

Twenty one codes and basic themes (8 for ART services, 3 for OST services and 10 shared for both services) emerged from the transcripts. The basic themes were then categorised into three organizing themes based on the framework used for the quantitative analysis for convergence and synthesis of the findings. The three organizing themes were–individual level, health system level and structural barriers. Fig 2 depicts a web-like, non-hierarchical illustration of the basic and organising themes around the global theme of barriers to accessing ART and OST services.

#### Individual level barriers

Incorrect and insufficient knowledge about the ART services and myths related to antiretroviral drugs and especially about the side effects and long term consequences of ART were expressed as a reason for not accessing ART. “IDUs have a misconception that this medicine is not useful for them, it causes illness to them and we have side effects. When he leaves the medicine in between, he dies.” said a peer-PWID. As it was also shown in the quantitative analysis, PWID were more knowledgeable about OST drugs and services than for ART.

Several access barriers were shared among ART and OST services and especially the high mobility among PWID. Many PWID are migrants and highly mobile and this prevented them from either accessing services or, once registered, from regularly attending these services. “Biggest problem is mobility; after testing, they move to different locations” said a TI counsellor.

Interviewees often reported low self-esteem and even more often attributed the lack of enrolment to health services to the high drug dependence and the drug use itself: “They will not go, as they are badly addicted to drugs” said a peer PWID himself.

#### Health system level barriers

“Two of my clients died during the testing process of ART. If their medicine would have started on time irrespective of tests, they might have been saved” a TI programme manager who has personally accompanied some clients to ART and OST services has said. Long delays in initiation of treatment, either antiretroviral or opioid substitution, was seen as a major barrier in retaining patients in care. Health providers from both services shared that funding gaps and long procedures in initiating therapy could be also considered as an access barrier and this may go as “word of mouth” among PWID. Importantly, if ART and OST were offered as a “one window service” the uptake of both would have been greater.

Overall, the OST services seemed to better address the needs of their clients. The main issues emerged was the lack of OST doctors and the consequent delays in treatment initiation. The PWID and the staff said that the timing of public OST was not optimal for the clients while the most important challenge was the occasional OST drug ruptures. In some instances the “drug dosage has to (be) halved…” and this was perceived as unacceptably bad practice by both providers and users.

The ART centres were not at all tailored to the needs of this key population. The timing of the centres was major barrier expressed by the PWIDs as well as the TI NGO and other staff. They also have a rather strict requirement of identity documents as well as proof of address which emerged as a major barrier for PWIDs.

In addition to the short and inflexible opening hours, long waiting times, overcrowded waiting bays and a judgmental, stigmatizing and unkind attitude of some ART personnel made the ART services unattractive and even hostile to PWID.

“They don’t like us because most of us leave the medicine in-between” said a PWID about the ART centre staff. In an extreme case, captured in one of the interviews, a PWID was physically abused (“beaten up”) by an hospital guard because “… he was a drug addict who was trying to bathe at the toilets…”.

#### Structural barriers

Homelessness, lack of family support and poverty were important challenges expressed by providers as well as PWIDs for accessing health services in general and ART and OST services in particular. As an outreach worker from a TI NGO pointed out “If they are homeless then we have to try hard to reach them”. Transportation costs, loss of income during long waiting hours and lack of food were reported as barriers and almost every interviewee at some point during the interview mentioned food as an essential intervention. Nutritional support would not only make the ART and OST services more attractive to PWID but it could contribute to the health and wellbeing of these people, especially the extremely poor and homeless.

Stigma towards the PWID regarding their IDU and HIV status was a common barrier for both services, but especially for ART. “ART staff have perception that an IDU is a thief and steal the things from hospital.” a TI PM said, and continued: “According to them it’s worthless to start IDUs on ART medicine, as IDU will not continue it due to their drug habits and some day they will drop out.”

#### Suggestions for overcoming barriers

During the data collection key informants (and occasionally peer PWID) were spontaneously suggesting potential solutions to overcome the barriers being discussed. The interview guides also prompted for suggestions and recommendations during the interviews. All such suggestions were coded and categorised into individual, health system and structural solutions, separately for ART and OST services and are detailed in Table 2.

**Table 2:** Suggestions to address the barriers to accessing ART and OST services as perceived by key informants and PWID, Delhi (December 2016).

Barriers	ART	OST
Individual-level barriers	1. Drug detoxification and rehabilitation services should be provided to the PWIDs as a part of the programme.	1. OST should be started for PWIDs before initiating ART
	2. Biometric System should be introduced so that the PWID can access his / her ART and OST at any centre.	
Health-care system level barriers	1. A “single window” system should be adopted for all ART and OST services	1. OST timings should increase, or OST should be in two shifts for better coverage.
	2. ART centre timing should increase to accommodate the PWID schedules	2. IEC material with information about benefits of OST and ART services should be available
	3. Preferential treatment to be given to PWIDs by having a separate queue / separate time	3. Intensified outreach mechanism should be developed for referral and linkages.
	4. ART medicine should be started irrespective of their CD4 count	4. Peer leaders who are role models should be identified and peer group committees formed for facilitation of services.
	5. Provision of link ART center at TI level where there is more concentration of HIV positive PWIDs.	
	6. Intensified outreach mechanism should be developed for referral and linkages even beyond ART and OST services (e.g. food, shelter, social benefits, etc.)	
Structural barriers	1. Food coupon/token should be given to PWIDs to meet their nutritional requirement.	1. Involvement of family for treatment support
	2. Provision of shelter with basic amenities to maintain personal hygiene	2. Multi stakeholder involvement by advocacy with police and other local leaders.
	3. Involvement of family for treatment support	3. Provision of nutritional support, medical treatment for other ailments
		4. Skill development, education to be provided

## Discussion

This mixed methods study among homeless, HIV-positive PWIDs in Delhi revealed that while an unacceptably small proportion of them were on ART, a majority were aware of and availing OST services. Inconvenient timings, long waiting times, stringent registration requirements and negative attitude of health providers were major barriers of accessing ART services while these were not a concern in OST services. Homelessness, poverty, stigma were common barriers for both services. Integrated, “single window” service and provision of additional support like nutrition and shelter were suggested as measures to improve access by both health providers and the PWIDs themselves.

An unexpected finding of this analysis, which was not part of the primary and secondary study objectives, was that more than one third of the PWID included in the study first injected drugs when they were less than 18 years old. This alarming finding highlights the need for urgent comprehensive interventions to prevent drug use among adolescents and children, especially among street children, as well as to reduce harm among the ones who have already started using injectable drugs.

Globally, PWID are disproportionately less likely than are other HIV-positive patients and other key populations to receive ART. This holds true even in the five countries with the largest HIV epidemics concentrated among PWID (China, Vietnam, Russia, Ukraine, Malaysia); in these countries PWID were 67% of HIV cases and only 25% of those receiving ART [17]. In a study done in 2012 in Northeast India, which is the epicenter of the HIV epidemic among PWID in India, the ART coverage was 5% while it was 12% in our study which is aligned with the global reported range from 2% to 18% [18–19]. Similarly, OST registration among this group was very high at (80% as compared to North East India 21% and that at the national level at 32% [18].

The top three barriers in accessing ART by male PWIDs in northeast were travelling distance, fear of being identified by others as HIV-positive and the need to take care of others at home. The top three barriers to OST services were travel distance from the centre, fear of being identified by others as a drug user, and fear of being harassed by police [18]. These barriers were of a very low concern to the PWID in this study as they were not applicable in the urban metropolitan city of Delhi among the homeless population. In a study among PWIDs in Chennai active drug use, multiple stigma related to drug use, HIV-positive status, and homelessness were the barriers to ART services which were similar to our findings [10]. This highlights the need to constantly and dynamically study the targeted key populations, their demographic and social characteristics as well as the perceptions of providers and users in order to optimize the uptake of the offered services.

Stigmatization of IDUs in health settings, hidden or collateral fees, and multiple requirements for treatment initiation have been described as the main systemic barriers in a review among the five high-burden countries which also emerged in our study [17]. However, in Northeast India, discrimination against the PWIDs did not emerge as a barrier either for ART or OST services [18]. Drug shortages leading to delays in initiation and treatment interruptions at the OST centres were seen in other countries like Kyrgyzstan, Moldova, and Azerbaijan also [20]. The qualitative data also shows that there are occasional interruptions in the supply of the OST drugs at the OST centres which results in reducing the dose of the drugs given to the PWID. This practice is not considered good by the staff as well as the PWID. Another study conducted in Delhi also shows a similar result that a PWID requires an adequate dose of drug to give the required relief. A gap in the demand and supply can result in diversion from OST [21]. Another review and meta-analysis shows that lack of adequate dose of OST is also a challenge in scaling up the OST program apart from other barriers [22].

Insufficient knowledge about ART was found to be one of significant barriers in ART uptake which needs to be addressed. A study done in TI program for PWIDs at different places in India shows that the PWIDs have good knowledge about ART but they might lack the in-depth knowledge of ART. The incomplete/no knowledge about the side effects of the ART drugs is also a challenge [12]. A similar study conducted in South India also indicates that insufficient knowledge about ART also leads to lack of adherence to the ART treatment [23].

An overarching theme that emerged from the data during the qualitative analysis was that the OST services were more accessible, user-friendly and tailored to the needs of the PWID. Even though such a finding was somehow expected, as OST are by definition targeted, specialized services, this was a view shared and confirmed by the PWID interviewees. Moreover, it was revealed by the different language, terminology, tone and overall attitude of the interviewed OST staff who showed respect, engagement, motivation and an expressed wish to improve their services. The fact that some of the OST services staff were rehabilitated PWID may explain a difference in understanding, involvement and attitude between OST and ART staff. In any case it was felt again during the analysis that ‘single window services’ with flexibility of timings would have been ideal in addressing several of the barriers emerged from the quantitative and qualitative data. Similar changes have been suggested for PWIDs in Chennai [10,24]. Intensified outreach activities through NGOs for providing ART and OST services, provision of additional benefits like food, shelter, drug detoxification and rehabilitation, and social benefits have been suggested as probable ways of overcoming some of the major barriers.

Starting HIV positive PWIDs on ART irrespective of their CD4 count as soon as the diagnosis of HIV is made came as a strong suggestion and is supported by the latest evidence and the latest WHO guidelines [25]. This is already in the pipeline as a part of the modified strategy of the NACO based on the WHO ART Guidelines 2016 and will be implemented soon.

The continuous struggle of the homeless PWIDs with the police, administration and their legal status predispose them to multiple encounters with the police department. Sensitisation of and advocacy with the police department were also suggested as measures to improve access. Similar measures have been suggested in other studies [17].

Our study had several strengths. Firstly, we employed a mixed methods design which gave comprehensive and complementary views of the perceived barriers to ART and OST services, both in quantitative and qualitative terms. Secondly, we used a validated questionnaire and standardized operational definitions and data collection methods. Thirdly, as quantitative data were collected prospectively, missing data were minimal. Fourthly, there was a very high acceptance of the quantitative interviews among PWID at 78.6% and this had a positive effect on the study validity and generalizability of the findings. Lastly, we adhered to the STROBE guidelines and the consolidated criteria for reporting qualitative research (COREQ) guidelines [16, 26]. However, our study had some important limitations as well. Firstly, quantitative data were self-reported and this might have limited the validity, generalizability and transferability of study findings. Secondly, we relied on routine programme records from multiple TI projects to draw our study participants and this might have influenced the representativeness of the study sample. However, as the participating TIs covered 81% of the total Delhi PWID estimated population we believe that our sample provided robust finding. However we acknowledge that some of the most deprived homeless PWID, especially the ones “in transit” may go totally “under the radar” of even the most comprehensive outreach programme and such marginalized sub-populations are likely to be understudied and underserved. Limitations notwithstanding, this mixed methods study provided useful insights into the main barriers in accessing ART and OST services among homeless HIV-positive PWID, one of the most marginalized key populations in Delhi.

In conclusion, we note the urgent need for structural and health systems changes to improve access to ART and OST, including, integrated service delivery from a “single window”, accelerated ART initiation, simplification of bureaucratic procedures and nutritional and social support to all homeless PWID in this setting. We also note the urgent need for early preventative interventions among adolescents and street children.

## Supporting information

S1 Data Set (XLS)

S1 Data Code Book (XLSX)
